# Preclinical success but clinical failure of the sutureless excimer laser-assisted non-occlusive anastomosis (SELANA) slide

**DOI:** 10.1007/s00701-018-3686-6

**Published:** 2018-10-02

**Authors:** T. P. C. van Doormaal, B. de Boer, S. Redegeld, S. van Thoor, C. A. F. Tulleken, A. van der Zwan

**Affiliations:** 1 Department of Neurosurgery, University Medical Center Utrecht, Utrecht, The Netherlands; 2 Brain Technology Institute, Utrecht, The Netherlands

**Keywords:** Anastomosis, Ec-Ic bypass, Neurosurgery, Neurosurgical devices, Preclinical

## Abstract

**Background:**

The excimer laser-assisted non-occlusive anastomosis (ELANA) has been developed for intracranial bypass without the need for temporary recipient occlusion. We designed and tested a sutureless variant of the ELANA—the SELANA slide (SEsl).

**Objective:**

This study aims to evaluate the SEsl preclinical results and describe its first clinical application.

**Methods:**

First, in a cadaver study, 28 SEsl anastomoses were compared with 28 ELANA anastomoses. Second, in an acute rabbit model, 90 SEsl anastomoses were compared with 30 ELANA anastomoses. Finally, in a surviving pig model, 38 SEsl bypasses were created. To evaluate the clinical efficacy of the SEsl, we then treated one patient with a giant, right-sided middle cerebral artery (MCA) aneurysm with an intracranial–intracranial SEsl bypass and parent vessel occlusion.

**Results:**

In preclinical studies, the SEsl anastomosis was shown to be equivalent or superior to the ELANA in terms of associated ease, patency, and bleeding complications. However, clinical application in rigid and arteriosclerotic receiving arteries was problematic. Although bypass creation and aneurysm occlusion were technically successful and the patient was postoperatively well, a pseudoaneurysm formed postoperatively at the internal carotid artery anastomosis and bled. Subsequent treatment failed and the patient did not survive.

**Conclusion:**

The SEsl showed promising preclinical results across three models. However, in its present form, it is not suitable for clinical application.

**Trial number:**

IRB UMCU 10/154.

## Introduction

**Fig. 1:**
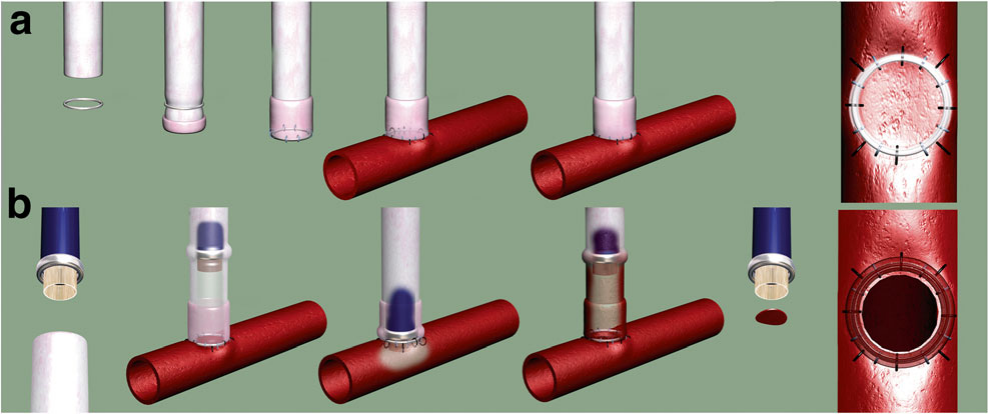
The ELANA technique (reprinted with permission [4]). **a** A 2.8-mm platinum ring is attached to the bypass graft with eight microsutures (size 8/0). Mostly, the saphenous vein is used as bypass graft, though use of the radial artery, a donor cadaver vessel, or an artificial vessel is also technically possible. Subsequently, the ring with graft is attached to the recipient artery with eight microsutures (size 8/0). The first four sutures, one each at the north, east, south, and west sides, are placed around the ring and through the recipient vessel wall. Four subsequent sutures are made through the bypass graft tissue outside the ring and through the adventitial and medial layers of the recipient artery. The recipient artery is not temporarily occluded during this attachment. **b** The ELANA catheter is inserted into the bypass graft. After 2 min of vacuum application, a disc of recipient artery wall (the “flap”) is lased from the anastomosis for 5 s at 40 pulses/s at 10 mJ/pulse. This opens the anastomosis without temporary occlusion of the recipient artery. The punched-out flap is then fixed to the tip of the catheter by vacuum suction and retracted together with the catheter. A temporary clip is then placed on the bypass graft, completing the anastomosis

The excimer laser-assisted non-occlusive anastomosis (ELANA) was developed for the creation of intracranial bypasses without a need for temporary occlusion of the intracranial artery and thus minimizing risk associated with occlusion time (Fig. 1). This technique has now been performed in over 500 patients worldwide, mostly for the treatment of complex and giant aneurysms [2, 3, 6].

Despite recent advances in endovascular techniques, a continuing search for improving available neurosurgical techniques and broadening their applications to more complex surgical cases remains valuable.

A major disadvantage of the classical ELANA technique is that a minimum of eight microsutures are required to fix the device’s platinum ring to what is often a deep intracranial vessel (e.g., the internal carotid artery (ICA) or proximal posterior cerebral artery (P1)). Even with the high-level of microsurgical skills required, the total suture time can vary from 45 min to 2.5 h [4]. The exact placement of these sutures is also essential for symmetry in tension of the recipient vessel wall within the ring. Any flaws may lead to damage to surrounding tissues or unsuccessful lasing resulting in poor retrieval of a full-thickness flap from the arterial wall. Proper surgical training is therefore critical, as there is a significant learning curve associated with learning the technical skills necessary for this procedure.

Between 2004 and 2010, our group developed a new “sutureless” ELANA (SELANA) [4, 5, 7]. It was our expectation that by reducing the number of deep intracranial microsutures necessary to attach the ELANA to the appropriate vessel, we would reduce suture-related complications and surgery time. In addition, by simplifying and standardizing the attachment procedure, the anastomosis could theoretically be performed in anatomical locations where securing sutures in one or more quadrants is impossible, as often occurs in very large, space-occupying aneurysms.

**Fig. 2:**
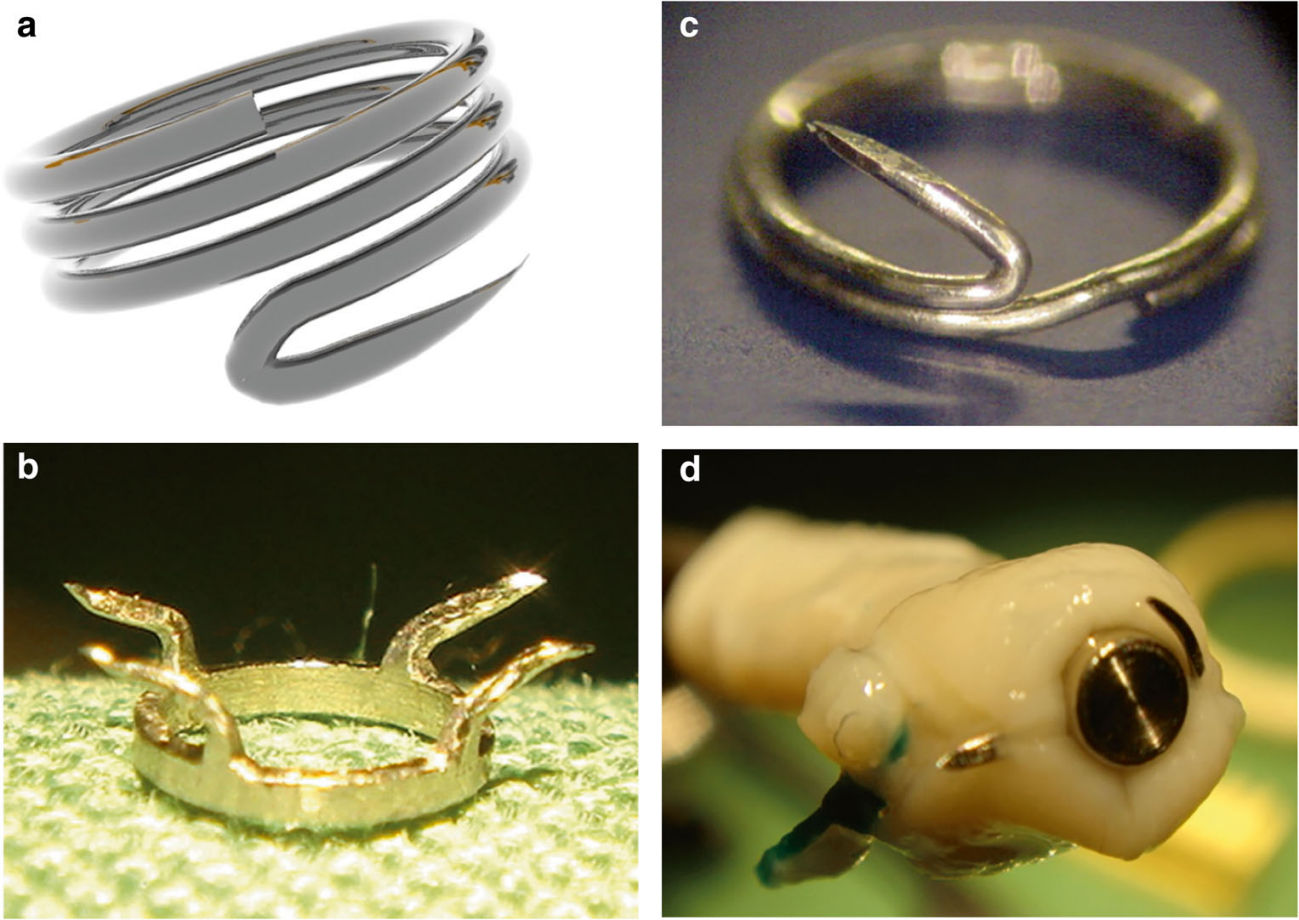
Examples of failed SELANA rings. Experimental application in vitro of these devices led to leakage or tears in the recipient vessel wall. **a** SELANA “screw,” **b** ELANA with four pins, **c** modified SELANA screw, **d** ELANA ring with straight and curved pin

During its development, we created many prototypes of the SELANA (Fig. 2). In 2009, the final iteration—the SELANA “slide”—was designed. The objective of this report is to evaluate and review preclinical SELANA slide (SEsl) studies and its first application to human neurosurgery.

## Methods

All work, up to and including the first clinical application of the SELANA slide, is described here. Part of this research was previously published [2, 3, 5, 6].

### SELANA slide design

**Fig. 3:**
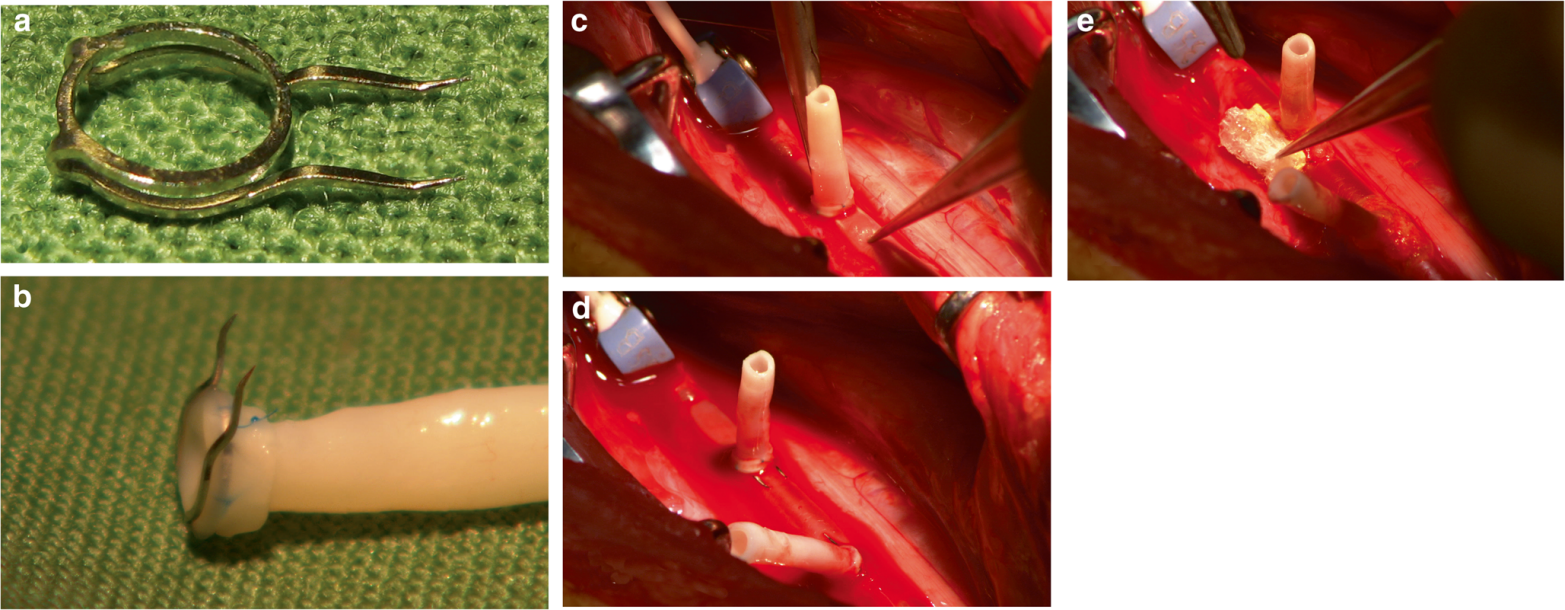
SELANA slide insertion and sealing (reprinted with permission [2]). **a** The SELANA slide ring. **b** The attachment of the SELANA ring to the donor vessel. **c** The slide is introduced to the recipient vessel, using a needle holder at the back. **d** The slide in place. The curves of the pins position the upper ring after application (puncture from the outside-inwards then after horizontal translation inside-out) flush with the recipient vessel wall. **e** The anastomosis is sealed with Tachosil (Takeda, Osaka, Japan)

In 2009, the original ELANA ring was modified to feature two sharp pins that eliminated the need for intracranial sutures. We designated this device the SELANA “slide” (Fig. 3a). The slide was built manually in our laboratory with surgical-grade steel (type 316L 1.4441) and tested according to ISO 5832-1:2007 “Implants for surgery” (https://www.iso.org/standard/39023.html) and to ISO 10993-1 “Biological evaluation of medical devices” (https://www.iso.org/standard/44908.html). It was also tested according to the applicable American Society for Testing and Materials (ASTM) criteria (F2052, F2213, F2182, and F2119) This included standard fatigue tests and MRI compatibility tests.

### SELANA slide technique

In short, first the slide had to be attached to a donor vessel (Fig. 3b). This procedure is performed outside the cranium. Mostly, the saphenous vein (SV) is used; however, the use of the radial artery or an artificial vessel is theoretically also possible. Subsequently, the slide including donor vessel is attached to the recipient artery (Fig. 3c). The minimal diameter of this recipient artery is 2.5 mm. Both pins are then simultaneously punctured through the recipient wall from outside-inwards. The slide is then horizontally pushed into the vessel. When the straight portions of both pins are fully inserted into the recipient vessel, they puncture the vessel wall again, but now from the inside-out. The slide can then be further inserted horizontally until a click is felt and it is in position (Fig. 3d). This fixes and slightly stretches the recipient artery wall within the circumferential shape of the pins. The SELANA slide anastomosis is then sealed with Tachosil (Takeda bv, Osaka, Japan) (Fig. 3e). Subsequent opening of the anastomosis with the laser catheter is identical to the conventional ELANA anastomosis (Fig. 1b).

### SELANA slide in vitro test

Four pressurized human cadaver heads were bilaterally trepanated, using a combined pterional–pretemporal–transcavernous approach [5]. In each head, seven ELANA anastomoses and seven contralateral SELANA anastomoses were constructed at different sites in the anterior and posterior circulatory systems, including in the ICA, MCA, anterior cerebral artery (ACA), and posterior cerebral artery (PCA).

### SELANA slide acute in vivo test

In 20 rabbits, the abdominal aortas were used as recipient arteries, paired with human saphenous veins as donor grafts, to make 30 conventional ELANA anastomoses with eight microsutures [3]. For comparison, 90 SELANA slide anastomoses were prepared. The SELANA slide anastomoses were combined with three different sealants: Tisseel (Baxter, Deerfield, Illinois, USA), Bioglue (Cryolife, Kennesaw, Georgia, USA), and Tachosil (Takeda Pharmaceuticals, Osaka, Japan). Outcomes for each procedure, including application time, associated complications, and burst pressure, were then compared.

This Dutch Central Animal Experimental Committee (DCAEC, https://www.centralecommissiedierproeven.nl) and the Animal Welfare Body Utrecht (AWBU http://www.ivd-utrecht.nl/en/) approved this study. The animal care complied with the Guide for the Care and Use of Laboratory Animals (GCULA, Institute of Laboratory Animal Resources, Commission on Life Sciences, National Research Council. Washington: National Academy Press, 1996, http://nap.edu/openbook.php?record_id=5140).

### SELANA slide chronic in vivo test

A bypass was performed on the left common carotid artery (CCA), using the right CCA as a graft, with two SELANAs in 38 pigs [2]. Pigs in both groups were maintained for up to 6 months, at which point an angiography was performed and the animals were euthanized. Scanning electron microscopy and histological studies were used to evaluate the anastomoses post mortem. Study results were compared with an earlier ELANA study performed in the same model [1]. The DCAEC and AWBU approved this study, and the animal care complied with the GCULA.

### SELANA slide patient

**Fig. 4:**
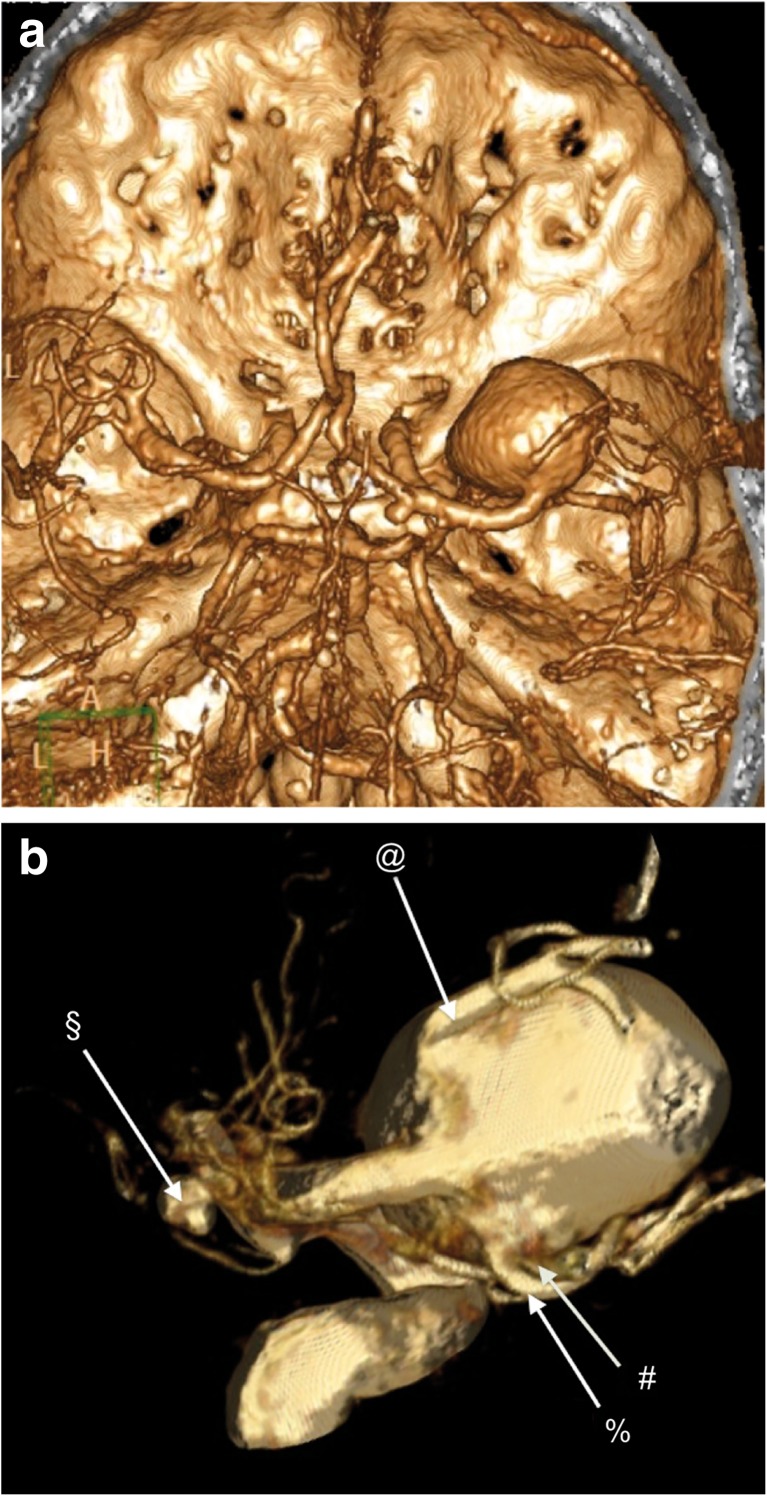
Preoperative imaging of the first SELANA slide patient. **a** CTA reconstruction of a right-sided giant MCA aneurysm. A second small distal ICA aneurysm can also be observed. **b** 3D DSA reconstruction of the right MCA aneurysm and emanating M2 branches. @, A larger MCA branch and later distal recipient of the IC-IC bypass; %, a smaller MCA branch; #, lenticulostriatal perforators from the aneurysm; §, small distal ICA aneurysm

Based on the results of the above studies, the medical ethical committee of our University Medical Center approved a pilot study to clinically assess the feasibility and safety of the SELANA slide technique in three patients. Written informed consent was obtained from the patient whose case is described here. The experimental protocol and informed consent were approved by our institutional review board (IRB, number 10/154). A 62-year-old woman presented with a giant, unruptured right-sided MCA aneurysm. She also had an asymptomatic, smaller distal right ICA aneurysm. The patient presented with signs of mass aneurysm effect (headache, slight paresis) and frequent thromboembolic events, most likely originating from the aneurysm sac. After thorough interdisciplinary evaluation with neurosurgeons, interventional radiologists, and neurologists, the aneurysm was determined to be a poor candidate for clipping or endovascular treatment (Fig. 4). Our surgical plan included an intra-to-intracranial (IC-IC) high-flow replacement bypass combined with parent vessel occlusion. We then planned to use one proximal SELANA slide anastomosis on the ICA and a distal SELANA anastomosis on one of the M2 branches. We planned to use the saphenous vein (SV) as interponate. Intraoperative flow was measured with a Transonic flowmeter (Ithaca, NY, USA).

## Results

The SELANA slide showed promising preclinical results, but caused a hemorrhagic complication in the first patient with whom it was used. This resulted in termination of our clinical study after one patient, though three were originally planned.

### SELANA slide in vitro test

The SELANA slide anastomoses, when performed in human cadaver heads [4], were shown to be feasible on the ICA, ACA, MCA, and PCA. The SELANA anastomoses were completed significantly faster than the ELANA anastomoses (mean time difference—11 min on the MCA; 107 min on the P1 segment of the PCA).

### SELANA slide acute in vivo test

The SELANA slide anastomoses, when performed in living rabbits [5], were constructed six times faster than conventional ELANA anastomoses (mean 2.5 ± 1.8 min vs. 14.8 ± 2.6 min, respectively). Flap retrieval rate was 93%, not significantly different from the ELANA flap retrieval rate (90%). The SELANA slide, when used in combination with Tachosil, resulted in no cases of intraoperative leakage of the 30 anastomoses performed. Moreover, all anastomoses showed a postoperative burst pressure above 280 mmHg.

### SELANA slide chronic in vivo test

The 76 SELANA anastomoses, when performed in living pigs [7], were created in a mean of 7 ± 2 min. Seventy-two devices (95%) were inserted in only one movement and four (5%) were re-applied after one of the pins failed to totally perforate the recipient vessel wall. All incidents of leakage were adequately controlled with gentle compression and there were no cases in which extra stitching or temporary vessel occlusion was needed. The flap retrieval rate in this model was 91%. Three anastomoses (4%) evidenced some leakage after lasing. This result was equivalent to the leakage rate reported in an earlier ELANA study in the same model [1]. In all three cases, sealant malposition was attributed to the leakage. Furthermore, all leakage stopped after extra sealant application and without any requirement for temporary occlusion. Mean bypass flow was 160 ± 56 cc/min. Upon follow-up, 33 of 38 bypasses were patent (87%), a percentage that fell within our predefined limits of non-inferiority to ELANA. Furthermore, no pseudoaneurysms were discovered. Complete endothelialization was observed after approximately 3 weeks in all cases.

### First SELANA slide patient

The senior author (AvdZ) was the first surgeon and the first author (TvD) was the second surgeon in the case described here. A pterional approach with subsequent dissection of the aneurysm and surrounding vessels, as well as harvesting the SV, was conducted without complications. The ICA had a diameter of 5 mm and the proximal MCA had a diameter of 3 mm. The flow through the proximal MCA was 35 cc/min. The biggest distal M2 branch (Fig. 4) had a diameter of 3 mm and a flow rate of 22 cc/min. These three vessels were all considered to be suitable for the SELANA slide. After harvesting the SV, a part of this vein was positioned into the slide without problems, as described in Fig. 3b.

Subsequently, the first SELANA slide, including the donor vein, was slid into the wall of the proximal M1. However, this was complicated. The vessel was significantly more rigid than the in vivo experimental vessels. A small vessel rupture occurred at the insertion point of one of the pins. This location was therefore abandoned, leaving the slide in place to preserve the integrity of the MCA. The anastomosis was not lased, but rather an aneurysm clip was placed on the small remnant of the vein at the top of the ring to prevent further vessel leakage.

A second slide, including a donor vein, was placed more proximally in the ICA wall, just before its bifurcation. Here, it was required to apply more translational force to slide the vessel wall over the curvature of the pins and to bring the pins in-and-out than was typical in the in vitro and in vivo experimental surgeries. However, the slide was placed and lased twice, and this full SELANA slide anastomosis was performed within 6 min. The application of extra Tachosil was required to control some minor bleeding after lasing. After full hemostasis, a disc of recipient vessel wall (the so-called flap) was retrieved from the ELANA catheter tip as evidence of the anastomosis’s patency.

**Fig. 5:**
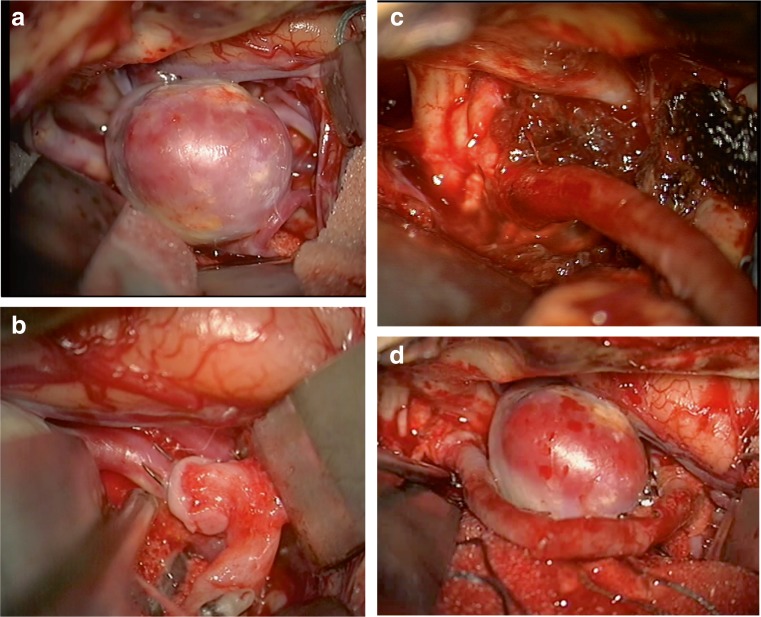
Intraoperative images**. a** Aneurysm exposed. **b** MCA SELANA slide anastomosis just after application before lasing. **c** ICA SELANA slide anastomosis after lasing with hemostatic material surrounding the anastomosis. **d** Completed bypass

Finally, a third SELANA slide was placed on the M2 and a complete anastomosis was performed within 4 min, including lasing twice (Fig. 5). The sliding movement in this instance also took some effort due to the same factors outlined above in the first two applications, though leakage was not encountered. Hemostasis was obtained directly after lasing and a flap was retrieved.

The following end-to-end anastomosis was made in 10 min (Fig. 5d). Flow through the bypass was 53 cc/min after the anastomosed M2 vessel, distal to the aneurysm and proximal to the clipped anastomosis, was occluded. With multiple clips placed, the aneurysm was subsequently occluded while perforators and the other M2 branch were kept open. During this process, the proximal ICA anastomosis started to bleed two times and was again managed with Tachosil. By the end of the procedure, the MCA aneurysm was completely occluded, leaving the other M2 and perforating branches open. The patient woke up without neurological deficits and recovered fast.

**Fig. 6:**
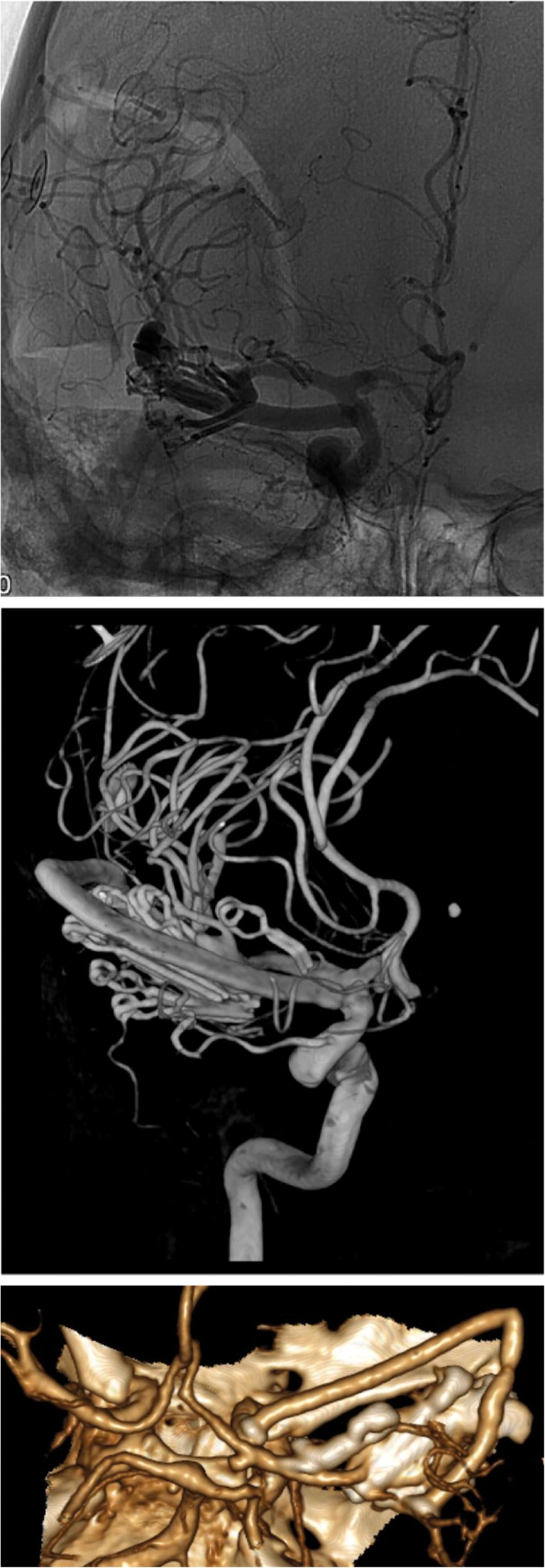
Postoperative imaging of ICA-M2 and IC-IC SELANA slide bypass. DSA (**a**), 3D angiography (**b**), and CTA (**c**) with a confined aneurysm is trapped. Left DSA: **#,** abandoned first SELANA slide; *, second SELANA slide on the ICA; **^,** third SELANA slide on an M2 branch

**Fig. 7:**
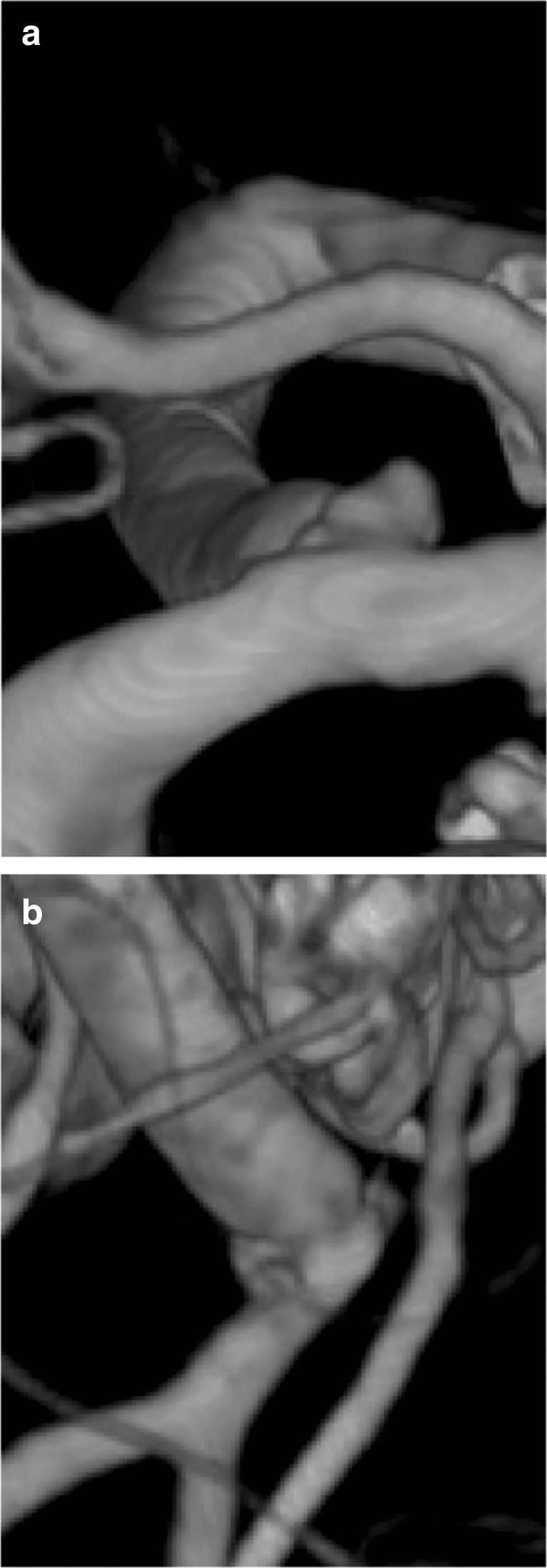
Postoperative 3D DSA. **a** Proximal SELANA slide anastomosis with pseudoaneurysm (arrow). **b** Distal SELANA slide anastomosis, directed at aneurysm, aneurysm thrombosis. No anastomotic irregularities evident

Initial postoperative 3D digital subtraction angiography (DSA) and CTA showed the IC-IC bypass to be patent and the aneurysm closed (Fig. 6). However, 3D DSA 14 days after surgery revealed a false aneurysm at the proximal segment of the bypass (Fig. 7). Due to this, we decided to re-operate on the patient, which took place 18 days after the first surgery. Treatment of this false aneurysm was attempted by placing a stitch at the back of the anastomosis. This was a technically challenging procedure. Continuous, heavy bleeding at the backside of the anastomosis resulted in blind placement of repair stitches. Full intraoperative hemostatic control was achieved. However, a new postoperative subarachnoidal and intracerebral hematoma developed 3 days postoperatively and the patient was lost.

**Fig. 8:**
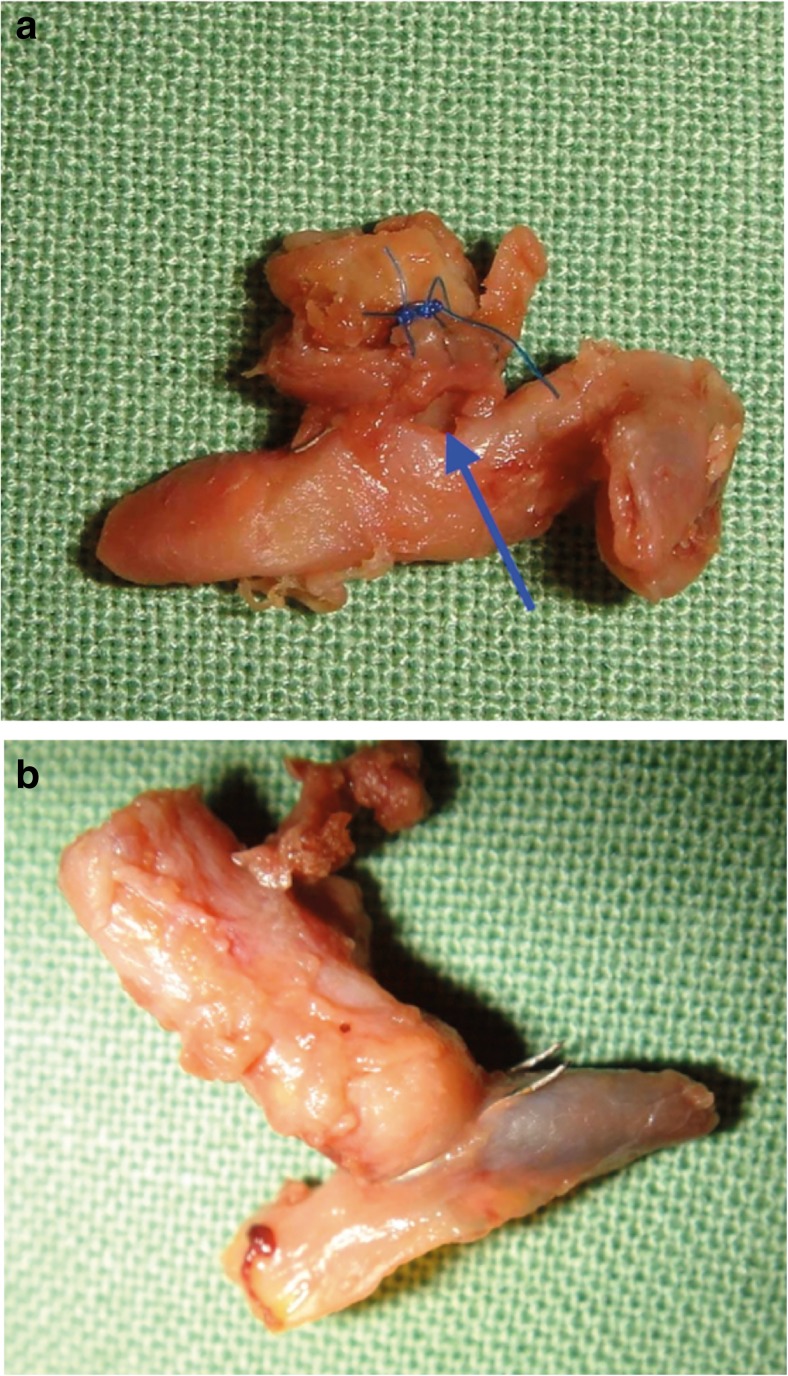
Anastomoses at obduction. **a** Pseudoaneurysm opening under the SELANA slide (arrow). Blue stitches applied during the second surgery are visible above the pseudoaneurysm. **b** Distal SELANA slide anastomosis

Upon postmortem examination, we found that the massive rebleed originated from the proximal ICA anastomosis where a false aneurysm formed. It became clear that the SELANA slide was not fully translated horizontally into the ICA after the initial vessel wall puncture, resulting in the development of a false aneurysm at the backside of the anastomosis (Fig. 8a). Unfortunately, stitches placed during the second surgery were not placed deeply enough to completely restore the false aneurysm. The bypass and distal SELANA slide anastomosis on the M2 branch were patent and did not show other abnormalities (Fig. 8b).

## Discussion

Although good results were obtained in the preclinical studies described here, application of the SELANA slide device failed upon application in our first patient. We believe it is important to show and discuss these results as they provide for unique learning opportunities and form a basis for future neurosurgical developments.

In human vessels with signs of arteriosclerotic change, horizontal translation of the SELANA was demonstrated to be more difficult and hazardous than in healthy animals. The SELANA ring pins were designed to perforate the recipient vessel wall. In our preclinical animal studies, some minimal resistance was encountered upon device insertion as recipient vessel walls were compressed between the ring and pins during its translation over the circular, laterally extending part of the pins. However, this resistance was in animals easily overcome by gentle translational pressure. A felt “click” indicated that the device was in place, providing the surgeon with tactile confirmation that the device’s installation had been performed completely and correctly.

In healthy animal vessels with fully intact elasticity, compression within the slide was feasible without resultant bleeding because of the slight lateral stretch caused by the round pins. It is remarkable that in our perfused cadaver study, the same technique also functioned very well and ruptures did not occur. However, this postmortem application of the SELANA slide study was performed with artificial donor vessels and an internal applicator that could not be used in patients. It is possible that a fresh cadaver study of the SELANA slide, with the SV serving as a donor vessel, or animal models featuring arteriosclerotic disease might have served as better experimental models.

In addition to a more difficult application and different donor vessel qualities, other differences were present between the preclinical animal models and the patient application described here. The first difference noted was that application of the device in the patient disturbed complete surgical oversight. It is possible that our use of animal models could have been more realistic when conditions of less oversight had been created (i.e., surgeon positioning over an operative field with only a small viewing window). Also for this reason, more realistic experimental human cadaver studies seem essential. An additional difference between animal models and the patient application described here was that the anastomoses in our patient after lasing were directly exposed to a higher mean arterial pressure than that which was implicated in animals (a MAP differential of approximately 20 mmHg). Although theoretically a fibrous layer could have formed in animals before the real burst pressure testing, this seems an unlikely explanation because the pressures simulated in vitro after harvesting of the anastomoses were excessively high (above 260 mmHg).

A point of retrospective consideration was whether the pseudoaneurysm might also potentially have been treated with a stent. However, subsequent anticoagulation protocols may have resulted in other problematic sequelae.

The unacceptable results of the first surgical application of the SELANA slide indicated that this device requires significant re-design and improvement. A new SELANA device should allow for improved sliding, better overview, avoiding compression, and easier pin insertion. In addition, this revised design should avoid double perforation of the recipient vessel wall and the need for the traction. The pins in this revised device should be free at the insertion point, allowing for more easily controlled and visualizable vessel wall penetration. Theoretically, this could prevent the clinical complication encountered in the case described here. To establish the clinical safety and efficacy of future SELANA slide designs, preclinical studies must be repeated with an emphasis on practical application and visualization.
